# Palytoxin-Containing Aquarium Soft Corals as an Emerging Sanitary Problem

**DOI:** 10.3390/md14020033

**Published:** 2016-02-04

**Authors:** Marco Pelin, Valentina Brovedani, Silvio Sosa, Aurelia Tubaro

**Affiliations:** Department of Life Sciences, University of Trieste, Via Valerio 6, 34127 Trieste, Italy; mpelin@units.it (M.P.); valentina.brovedani@phd.units.it (V.B.); ssosa@units.it (S.S.)

**Keywords:** palytoxins, *Palythoa*, *Zoanthus*, dermotoxicity, inhalational toxicity, aquarium

## Abstract

Palytoxin (PLTX), one the most potent marine toxins, and/or its analogs, have been identified in different marine organisms, such as *Palythoa* soft corals, *Ostreopsis* dinoflagellates, and *Trichodesmium* cyanobacteria. Although the main concern for human health is PLTXs entrance in the human food chain, there is growing evidence of adverse effects associated with inhalational, cutaneous, and/or ocular exposure to aquarium soft corals contaminated by PLTXs or aquaria waters. Indeed, the number of case reports describing human poisonings after handling these cnidarians is continuously increasing. In general, the signs and symptoms involve mainly the respiratory (rhinorrhea and coughing), skeletomuscular (myalgia, weakness, spasms), cardiovascular (electrocardiogram alterations), gastrointestinal (nausea), and nervous (paresthesia, ataxia, tremors) systems or apparates. The widespread phenomenon, the entity of the signs and symptoms of poisoning and the lack of control in the trade of corals as aquaria decorative elements led to consider these poisonings an emerging sanitary problem. This review summarizes literature data on human poisonings due to, or ascribed to, PLTX-containing soft corals, focusing on the different PLTX congeners identified in these organisms and their toxic potential.

## 1. Introduction

The history of palytoxin (PLTX) is closely connected to soft corals since the time of the Hawaiian *Limu-make-o-Hana* legend, which literally means “The toxic seaweed of Hana”. This legend tells of a man carrying a shark mouth on his back, used to kill fishermen entering in his fishing area. The man was killed by one fishermen that, after burning his body, threw his ashes into a tide pool near the harbor of Hana where, shortly after, started to grow a “toxic algae”. The warriors used to dip the tips of their spears in this water to make them fatal. At the beginning of 1960s, Prof. Helfrich discovered the exact location of this place, as well as the “toxic algae”, found to be a soft coral belonging to the genus *Palythoa* (*P. toxica*). Thus, the toxin identified in this zoanthid ten years later by Prof. Scheuer was called palytoxin [1]. Over the decades, chemical, biological, and toxicological studies on PLTX have elucidated the peculiar properties of this fascinating marine toxin that nowadays is considered one of the most toxic non-proteinaceous natural compounds.

After the beginning of its history, the interconnections between PLTX and soft corals have progressively lost their strength since the toxin and a series of its analogs have been subsequently identified in other marine organisms, phylogenetically very different from cnidaria, such as dinoflagellates, cyanobacteria, and edible vertebrates and invertebrates [2]. In particular, consumption of PLTX-contaminated seafood was associated with human cases of severe, and even lethal, adverse effects in tropical and subtropical areas. Subsequently, different toxicological implications for human health were ascribed to PLTXs in temperate areas: the signs and symptoms ascribed to these toxins involved mainly the upper respiratory tract and the skin, after inhalational, and/or cutaneous exposure to seawater and/or *Ostreopsis* cells concomitantly to these dinoflagellate blooms. Moreover, epidemiological data showed that the majority of the poisonings certainly ascribed to PLTXs could be linked to dinoflagellates, shifting the interest from soft corals to microalgae or to marine edible organisms that could accumulate these toxins through the food chain [2]. However, in recent years the toxicological implications of PLTXs for human health gradually recurred to the exposure through soft corals. Indeed, the number of case reports on human poisonings after manipulation of PLTX-contaminated soft corals, widely used as aquaria decorative elements by aquarium hobbyists, is continuously increasing. The widespread phenomenon, also due to the lack of coral trade controls, and the entity of the adverse effects led to consider these poisonings an emerging sanitary problem, even though still underestimated.

This review will summarize and discuss the documented cases of human poisonings due, or ascribed, to PLTX-contaminated aquarium soft corals, considering the different PLTX congeners currently identified in these cnidarians and the relevant toxicological potential.

### 1.1. Palytoxin: Producing Organisms

PLTX has been identified in a variety of marine organisms in tropical, subtropical, and temperate regions. The original source of PLTX was a soft coral, *Palythoa toxica*, collected in Hawaii. Throughout the years, it has also been identified in other species belonging to the genera *Palythoa* and *Zoanthus* [3]. More details will be given in Section 3.1, describing PLTX analogs identified in *Palythoa* and *Zoanthus* soft corals.

In 1995, a PLTX-like molecule was identified in benthic dinoflagellates of the genus *Ostreopsis*. This compound, isolated form *O. siamensis*, was named ostreocin-D (Ost-D; see Section 1.2) [4]. Interestingly, only *O. siamensis* of the Japanese strain was shown to produce Ost-D, and the toxin has never been identified in *O. siamensis* of the Mediterranean area, so far [5]. Other *Ostreopsis* species were later found to contain PLTX-like compounds: *O. mascarenenesis*, containing mascarenotoxins [6], and *O. ovata*, a source of ovatoxins (see Section 1.2) [7].

To explain PLTX’s presence in phylogenetically-different species, some authors proposed bacteria as producing organisms and a possible common source of these toxins. With this respect, Frolova *et al.* [8], using anti-PLTX antibodies, detected PLTX-like compounds in Gram-negative *Aeromonas* sp. and *Vibrio* sp. bacteria. Similarly, bacteria isolated from *Palythoa caribaeorum* were found to display a PLTX-like hemolytic activity [9]. In addition, PLTX and 42-hydroxy-PLTX were isolated from marine *Trichodesmium* spp. cyanobacteria [10]. However, a clear definition of the actual producing organism of PLTX is still a matter of debate.

### 1.2. Palytoxin: Molecular Structure

**Figure 1:**
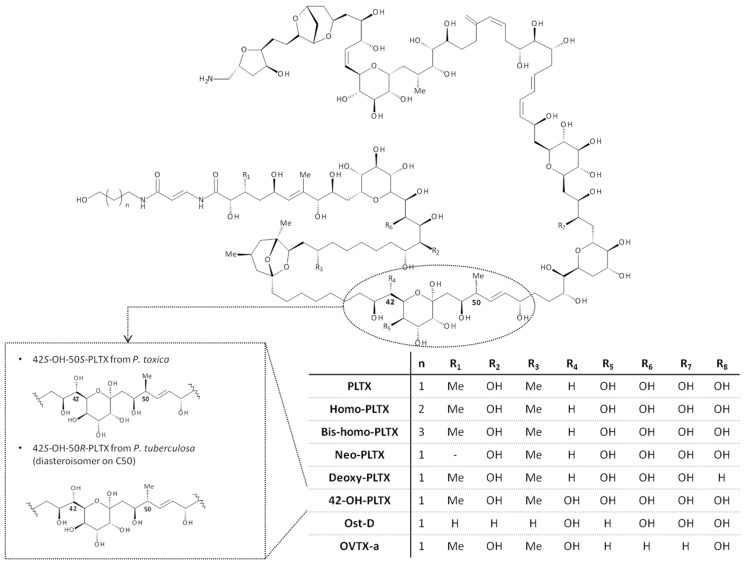
Chemical structure of PLTX and its main analogs.

The chemical structure of PLTX was elucidated in 1981, almost a decade after its first identification in *Palythoa toxica*, by two independent research groups [11,12]. The chemical formula of PLTX is C_129_H_223_N_3_O_54_, with a molecular weight of 2680.13 Da. PLTX is considered one of the most complex and large non-polymeric natural molecules: it contains 129 aliphatic carbon atoms, 40 secondary hydroxyl groups, two diene motifs, a conjugate acrylamide-enamide system, three unsaturated bonds, two hydrophobic hydrocarbon chains, cyclic ether systems, and bicyclic acetals (Figure 1). The structure contains 64 chiral centres, leading to a huge number of possible conformational stereoisomers [12]. Structural studies demonstrated that PLTX assumes a dimeric form in aqueous solution, acquiring a form of ∞, measuring 52.3 × 22.0 × 15.1 Å [13]. The PLTX moieties involved in the dimer formation have not been identified, so far, although the hydrophobic region (C21–C40) and the region around the conjugated double bonds (C60–C84) are thought to be tentatively involved. Moreover, the terminal amino group is probably involved in the interaction between the two PLTX molecules: its acetylation prevents the dimer formation and reduces the *in vitro* biological activity (smooth muscle contraction) by about 100 times with respect to PLTX [13].

In addition to PLTX, a series of its analogs has been identified, so far. They differ from PLTX for additional and/or missing hydroxyl and/or methyl groups, or for chiralities, which sometimes influence their toxic potency. Only few PLTX analogs have been studied under a chemical and/or biological point of view. Among them, two isomers had been isolated from soft corals: 42-hydroxy-palytoxin (42*S*-OH-50*S*-PLTX), isolated from *Palythoa toxica* [14], and its stereoisomer with a conformational inversion at C50 (42*S*-OH-50*R*-PLTX), extracted from *Palythoa tuberculosa* [15]. For a complete list of PLTX analogs identified in soft corals and the relevant toxicological properties, refer to Section 3.1.

The limited studies on PLTX analogs identified in dinoflagellates involved ostreocin-D (Ost-D), isolated from *Ostreopsis siamensis* in Japan [16], and ovatoxin-a (OVTX-a), the major toxin produced by *Ostreopsis* cf. *ovata* in the Mediterranean Sea [7]. Recent investigations identified several OVTX-a analogs (OVTX-b to -h) in *Ostreopsis* cf. *ovata*, at concentrations lower than those of OVTX-a. Intriguingly, in different parts of the Mediterranean Sea, OVTX-a has been always detected as the major *O. cf. ovata* toxin, with isobaric PLTX being frequently detected only in traces [7,17,18,19,20,21,22,23]. Very recently, an *in vitro* study demonstrated that OVTX-a cytotoxicity and binding affinity towards skin keratinocytes are more than two orders of magnitude lower than those of PLTX. Accordinlgy, also OVTX-a hemolytic effect seems to be lower than that of PLTX [24].

### 1.3. Mechanism of Action

The molecular target of PLTX is Na^+^/K^+^ ATPase, a transmembrane pump belonging to the family of P-type ATPases, essential for maintaining cellular ion homeostasis. Na^+^/K^+^ ATPase transfers three Na^+^ ions out of the cell in trade for two K^+^ ions, exploiting ATP hydrolysis. PLTX binding to the α-β ATPase heterodimer changes the transmembrane pump into a nonspecific monovalent cation channel, leading to a consistent ionic imbalance at the cellular level [25,26]. The channel formed by PLTX binding seems to be a consequence of ATPase conformational changes leading to a loss of pump gate control and uncoupling of the ion transport. In addition, PLTX binding reduces the rate of the pump de-phosphorylation, protracting the channel opening [25,26].

The cardioactive glycoside ouabain (OUA), known to inhibit Na^+^/K^+^ ATPase, has been reported to inhibit PLTX *in vitro* effects [27,28,29,30,31]. However, the incomplete abolishment of PLTX biological activities by OUA suggests that the latter does not completely compete with PLTX for the same molecular target [32]. In fact, Artigas and Gadsby demonstrated that PLTX and OUA can simultaneously bind to Na^+^/K^+^-ATPase, suggesting two binding sites on the pump [25]. Accordingly, the presence of a high-affinity binding site for PLTX on skin HaCaT keratinocytes was subsequently reported. This binding site appears to be partially insensitive to OUA and partially modulated by OUA in a complex manner: as a negative allosteric modulator against high PLTX concentrations (0.3–1.0 × 10^−7^ M) and as a non-competitive antagonist against low PLTX concentrations (0.1–3.0 × 10^−9^ M). This hypothesis could explain the inability of OUA to totally prevent PLTX-induced cytotoxic effects in HaCaT cells [32].

The transformation of Na^+^/K^+^-ATPase into a non-selective cationic channel by PLTX results in a sustained cellular ion homeostasis imbalance, as previously reviewed by Rossini and Bigiani [33]. The first event consists of an intracellular overload of Na^+^ causing a cell membrane depolarization, accompanied by a massive efflux of K^+^ and influx of Ca^2+^. Ca^2+^ influx seems to be mediated by reverse functioning of the Na^+^/Ca^2+^ exchanger (NCE) caused by the increased intracellular Na^+^ concentrations. Although not yet completely clear, the increased intracellular Ca^2+^ levels might induce the opening of K^+^ or Cl^−^ channels, further impairing the cell ionic balance. Moreover, the intracellular Na^+^ increase appears to induce a cytoplasm acidification, probably due to the reverse functioning of the Na^+^/H^+^ exchanger (NHE) [33]. It is widely accepted that the cytotoxic effects of PLTX are strictly dependent on this ionic imbalance. Depending on the cellular type (*i.e.*, non-excitable or excitable cells), Na^+^- or Ca^2+^-dependent cytotoxic effects have been reported. Na^+^ overload seems to be the first step in mediating PLTX-induced early cell damage, as recently demonstrated on human HaCaT keratinocytes [28]. Moreover, the intracellular H^+^ increase, consequent to the abnormal intracellular Na^+^ concentration induced by the toxin, seems to be the driving force for O_2_^−^ production by reversing the mitochondrial electron transport [34], ultimately leading to an irreversible necrotic cell death [35]. This finding is consistent with previous observations supporting Na^+^ dependency of PLTX effects [14,36,37,38]. On the contrary, in excitable cells, where intracellular signalling is highly dependent on Ca^2+^ concentrations with respect to non-excitable cells, PLTX effects are strictly dependent on Ca^2+^ ions. Indeed, the increased intracellular Ca^2+^ concentrations induced by Na^+^ overload were shown to trigger a series of Ca^2+^-dependent cytotoxic effects, such as neurotransmitter release, uncontrolled muscle cell contraction, up to Ca^2+^-dependent cell death [30,39,40]. As a secondary event to the sustained ionic imbalance, damages at the cytoskeleton level induced by PLTX, such as depolymerization of actin filaments in intestinal [38] and neuroblastoma cells [41,42], were also reported.

### 1.4. Human Risk Associated with Palytoxin Exposure

Cases of human poisonings ascribed to PLTXs have been generally associated with four exposure routes: (*i*) oral exposure; (*ii*) cutaneous exposure; (*iii*) inhalational exposure; and (*iv*) ocular exposure. The oral exposure after ingestion of contaminated fish or crustaceans is the most harmful for human health, although a limited number of foodborne poisonings have been documented only in tropical and subtropical regions, so far [43,44,45,46,47]. Among the foodborne poisonings ascribed to PLTX, only a few of them were certainly attributed to these toxins by their direct detection in the leftovers through biological and/or chemical methods of analysis. The vectors of PLTX were mainly crabs (*Demania reynaudii*; Alcala *et al.* 1988), parrotfish (*Scarus ovifrons*) [43], goldspot herring (*Herklotsichthys quadrimaculatus*) [45,46,47], and serranid fish (*Epinephelus* sp.) [46]. The symptoms of poisoning initially involved the gastro-intestinal tract (nausea, diarrhea, and vomiting) and the nervous system (convulsions, dizziness, numbness, and restlessness), with subsequent involvement of other excitable tissues, such as those of skeletal muscle (weakness, muscle cramps, myalgia, and rhabdomyolysis) and cardiovascular system (bradycardia, tachycardia). The clinical picture usually worsened, with symptoms involving the respiratory tract (rapid and shallow breathing, cyanosis, and dyspnea) leading, in some cases, to respiratory failure and death. Other documented cases were attributed to PLTXs by an indirect toxin detection in the causative species collected after or before the poisoning, sometimes even in different areas. Some cases were attributed to the toxin only on the basis of the clinical signs and symptoms associated with seafood ingestion. For a complete list refer to Tubaro *et al.* [2] and Wu *et al.* [47].

In temperate areas, human poisonings ascribed to PLTX were often associated with inhalation of marine aerosol and/or cutaneous exposures to seawater during *Ostreopsis* blooms. The most common signs and symptoms were respiratory distress, rhinorrhea, cough, fever, and dermatitis [48,49,50,51]. Several cases of adverse effects after exposure to seawater during *Ostreopsis* blooms occurred along the Italian coasts [48,51,52,53] and some episodes also along other Mediterranean coasts [49,50]. In these cases, the toxins detection and/or quantitation are often incomplete or missing, and they have been frequently ascribed to PLTX only on the basis of symptoms, anamnesis, and/or environmental data. Notwithstanding, the documented episodes could represent only the tip of the iceberg since these poisonings could be significantly underestimated. In fact, the symptoms do not always require hospitalization and could be frequently ascribed to a different etiology [2].

In recent years, there is growing evidence that inhalational and/or cutaneous exposure to PLTXs could also occur after handling PLTX-contaminated soft corals during maintenance of home marine aquaria. The toxic potential of PLTXs identified in soft corals, together with the uncontrolled trade of these zoanthids, raise a serious concern for human health. Due to the growing number of documented cases, these poisonings can be considered an emerging sanitary problem.

## 2. Human Poisonings Postulated to PLTX Exposure through Handling of Soft Corals

### 2.1. Exposure Routes

As reported above, adverse effects in humans ascribed to PLTXs have been generally associated with exposure to contaminated marine organisms and/or seawater through: (*i*) oral exposure; (*ii*) cutaneous exposure; (*iii*) inhalational exposure; and (*iv*) ocular exposure. Poisonings associated with handling of PLTX-contaminated soft corals are no exception, although ingestion of corals or surrounding seawater can be regarded as improbable, but not impossible, events. On the other hand, inhalation of vapors from home marine aquaria during the eradication of PLTX-contaminated zoanthids appears to be the most frequent route of exposure to PLTXs associated with soft corals. Indeed, since these corals rapidly colonize the aquaria due to the optimal growing conditions, they have to be frequently eradicated, usually by pouring boiling water and/or brushing the rocks carrying these cnidarians. The subsequent steam inhalation can induce a series of adverse effects involving the respiratory tract (*i.e.*, rhinorrhea, cough, dyspnea) but also other symptoms, such as myalgia, paresthesias, tachycardia, hypotension, fever, and gastrointestinal symptoms [1,54,55,56,57].

Similarly, accidental cutaneous exposure to PLTX-contaminated corals by aquarium hobbyists while cleaning marine aquaria has been associated with adverse effects. In addition to local inflammatory signs, such as edema and erythema, systemic symptoms of poisoning were experienced after handling the corals, both by intact or damaged skin. Among them, perioral paresthesia and dysgeusia were the most common ones and, in the most severe cases, transitory alterations of cardiac functions were also recorded [1,54,58].

Although ocular exposure is one of the less predictable exposure routes for PLTX, cases of eye irritation, mainly keratoconjunctivitis, occurred after the contact with mucous secretions from soft corals [59,60].

### 2.2. Human Poisonings Ascribed to Palytoxins-Contaminated Soft Corals: Direct Identification of PLTXs in the Corals

Similarly to the human poisonings associated with consumption of PLTX-contaminated seafood, documented cases of PLTX adverse effects by exposure to soft corals supported by the toxin’s identification in the causative specimens, are very limited (Table 1, Table 2 and Table 3). These cases will be grouped and reviewed on the basis of the exposure routes.

**Table 1:** Human poisonings due or ascribed to inhalational exposure to vapors or dust from PLTX-contaminated soft corals.

Location, Year	Number of Patients	Corals	Signs and Symptoms	Treatment and Outcome	PLTXs Detection Method and Concentration	Reference
Virginia (USA), 2007	1	*Palythoa/Protopalythoa* sp.	Foul odor. Difficult breathing, lightheadedness, chest pain, bronchoconstriction.	Anti-inflammatory corticosteroids and cough suppressant. Recovery after 1 month	Hemolysis neutralization assay (309 µg PLTX eq./g); HPLC (613 µg PLTX eq./g)	[54]
The Netherlands, 2014 *	4	*Palythoa heliodiscus*	Cough, dyspnea, chest pain, tachycardia, nausea. Leukocytosis with elevated neutrophils, CPK, CRP	Oxygen therapy, non-steroidal anti-inflammatory drugs. Recovery after more than 3 months	LC/MS (1018 µg PLTX/g wet coral; 46 µg 42-OH-PLTX/g wet coral)	[56]
Alaska (USA), 2014	3	*Palythoa heliodiscus*	Dyspnea, scratchy throat, paresthesia, myalgia, spasms, ataxia, weakness, tremors, nausea, tachycardia, fever	Supportive therapy. Recovery within 2 days	HPLC, LC/MS (7.3 mg PLTX/g wet coral)	[57]
Oklahoma (USA), 1961	3	*Palythoa caribaeorum*	Chills, nausea, headache	Recovery within 1 day	No experimental details (a compound identical to PLTX from *P. toxica*)	[1]
New York (USA), 2008 *	1	*Palythoa* sp.	Foul odor, shortness of breath, chest pain, sinus tachycardia	Inhaled albuterol. Recovery after 48 h	No analysis	[61]
The Netherlands, 2012 *	4	Zoanthids	Fever, hypotension, nausea, headache, shivering, muscle cramps. Leukocytosis, elevated CRP	Supportive therapy. Recovery within 48 h	No analysis	[62]
Switzerland, 2012 *	3	*Palythoa* sp.	Dyspnea, dry cough, nausea, headache, fever, chills, tachycardia, hypoxemia. Leukocytosis, slightly elevated LDH, CRP and procalcitonin. Restrictive ventilator pattern, diffuse bronchial swelling and secretion.	Treatment not reported. Recovery within 2 weeks	No analysis	[63]
New York (USA), 2013 *	5	*Palythoa* sp.	Shortness of breath, fever, dry cough, chills, myalgia, emesis. Leukocytosis, slightly elevated LDH, CPK, CKMB	Albuterol, levoflaxic, acetaminophen, hydration and supportive therapy. Recovery within 48 h	No analysis	[64,65]
Alaska, (USA), 2012–2014	9	Zoanthids	Bitter metallic taste, fever, tremors, weakness, ataxia, cough, joint and muscle pain, pulmonary symptoms	Treatment not reported. Recovery within 24 h, but sometimes with pulmonary symptoms after 2 years	No analysis	[57]
New York (USA), 2015 *	3	Zoanthid corals	Fever, chills, myalgia, tachycardia, wheezes, hemoptysis, dyspnea, leukocytosis, bibasilar opacities	Albuterol, acetaminophen, supplemental oxygen, prednisone. Complete recovery after 1 month	No analysis	[66]

* Year of publication; CPK = creatine phosphokinase; CKMB = creatine kinase MB isoenzyme; CRP = C-reactive protein; LDH = lactate dehydrogenase; eq. = equivalents.

**Table 2:** Human poisonings due or ascribed to cutaneous exposure to PLTX-contaminated soft corals.

Location, Year	Number of Patients	Corals	Signs and Symptoms	Treatment and Outcome	PLTXs Detection and Concentration	Reference
Hawaii (USA), 1962	1	*Palythoa toxica*	Dizziness, nausea, headache, malaise, discomfort to the hands	Supportive pharmacological treatment. Recovery after 1 week	NMR (280 µg PLTX/g wet weight)	[1]
Germany, 2008 *	1	*Palythoa* sp. and *Parazoanthus* sp.	Shivering, myalgia, weakness of the extremities, speech disturbance. Swelling and erythema at cut finger, numbness, and paresthesias of the arm. Slightly elevated CPK, LDH, CRP. Abnormal ECG	Infusion of intra-venous physiological fluids. Recovery after 48 h	Hemolysis neutralization assay (2–3 mg PLTX eq./g wet weight)	[58]
California (USA), 2009 *	1	Zoanthid corals	Metallic taste, perioral paresthesia, hives on torso and extremities, edema and erythema at hands. Urticarial rash on arms, things, abdomen, and back.	Intravenous diphenhydramine, methylprednisoline and lorazepam. Recovery after 24 h	No analysis	[67]
Georgia (USA), 2006	1	*Palythoa* sp.	Chest pain, lightheadedness, weakness, and numbness of the left arm, tachycardia. Elevated CPK	Supportive treatment. Recovery after 48 h	Patient serum: haemolytic activity, no neutralization by anti-PLTX antibody; no PLTX-like compound detection by HPLC, LC/MS	[54]

* Year of publication; CPK = creatine phosphokinase; CRP = C-reactive protein; LDH = lactate dehydrogenase; ECG = electrocardiogram; eq. = equivalents.

**Table 3:** Human poisonings ascribed to ocular exposure to PLTX-contaminated soft corals.

Location, Year	Number of Patients	Corals	Signs and Symptoms	Treatment and Outcome	PLTXs Detection and Concentration	Reference
N.D.	2	Zoanthids	Ocular irritation and redness, bitter metallic taste, eye pain photophobia, blurry vision, purulent discharge from eyes, bilateral punctate epithelial erosion, conjuctival hyperemia	Moxifloxacin, artificial tears, topical prednisolone acetate, fluorometholone, moxifloxacin, cyclosporine drops	Not performed	[59]
Switzerland, 2015 *	1	Zoanthids	Eyes burning, dyspnea, nausea, shivering, conjunctival injection, superficial punctuate epitheliopathy, multiple corneal Descemet’s folds, corneal erosion. Leukocytosis, elevated CRP, CPK, LDH	Intravenous infusion of balanced crystalloid solution, Diphoterine^®^, topical antibiotics and steroid, amniotic membrane transplantation, sclera contact lenses (4 months). Recovery within several weeks	Not performed	[60]

* Year of publication; CPK = creatine phosphokinase; CRP = C-reactive protein; LDH = lactate dehydrogenase.

#### 2.2.1. Inhalational Exposure to Vapors from Palytoxins-Contaminated Soft Corals

Cases of human poisoning associated to inhalation of vapors from hot water poured on PLTX-contaminated zoanthids, supported by PLTX detection in the involved cnidarian, are summarized in Table 1. The first well-documented case of a human poisoning by inhalation of steam from PLTX-contaminated soft corals was described in 2010 [54]. It involved a man in Virginia (USA) who eradicated from his aquarium a colony of green/brown medium-sized zoanthids, growing on live rock for three years. Pouring boiling water on the rock, the patient inhaled the steam and immediately felt a foul odor. Symptoms of poisoning, involving mainly the upper respiratory tract (rhinorrhea and cough), appeared within 20 min. The patient took an antihistamine agent, believing the symptoms could be caused by seasonal allergy. Notwithstanding, the symptoms (dyspnea and lightheadedness up to severe fits of coughing and chest pain) worsened within 4 h and the patient was hospitalized. Upon admission, his electrocardiogram (ECG) was regular, but it is unclear if hematochemical analyses were carried out. Pharmacological treatment was symptomatic with an anti-inflammatory corticosteroid and pain medications. After 15 h of hospitalization, the patient was discharged with prescribed inhaled corticosteroid and cough suppressant. A follow-up pulmonary examination, two weeks post-exposure, diagnosed asthma-like symptoms (bronchial inflammation and bronchoconstriction), so that the pharmacological treatment continued up to the complete relapse, one month post-exposure. Morphological analysis of the soft corals collected from the infested rock showed them as compatible with *Palythoa*/*Protopalythoa* sp. zoanthids. Hemolysis neutralization assay on the coral ethanol extract and high-performance liquid chromatography (HPLC) analysis determined 309 and 613 µg PLTX equivalents/g wet coral weight, respectively, whereas electrospray ionization-mass spectrometry (ESI-MS) confirmed that the toxin was consistent with PLTX. Subsequent investigations in a local Maryland aquarium store found a colony, morphologically consistent with the *Palythoa* sp. colony involved in the Virginia case, containing 515 µg PLTX equivalents/g, as determined by HPLC [55].

Recently, a poisoning due to steam inhalation from boiling water poured on a soft coral (*Palythoa heliodiscus*) involved four patients in The Netherlands. After patient 1 poured hot water over the coral, all patients developed cough (after 1–2 min) and dyspnea (after 5–10 min). The estimated exposure of patients 1–4 to vapors was 20, 15, 5, and 10 min, respectively, within six meters from the coral. The patients were admitted to the emergency room 45 min after starting the coral cleaning, with a series of symptoms including dyspnea, cough, chest pain, tachycardia, and nausea. Anamnesis showed that none of them had significant pre-existing health problems or smoking history. ECG and chest radiograph did not show any alteration, whereas hematological analyses revealed leukocytosis in all patients (15 to 34 × 10^3^ cells/µL) with high levels of neutrophils (patients 3 and 4: 31 and 15 × 10^3^ cells/µL), creatine phospho-kinase (CPK; patient 1: 215 IU/L) and C-reactive protein (CRP; patients 2, 3, and 4: 28–228 mg/L). The treatment was only supportive, consisting of oxygen therapy and non-steroidal anti-inflammatory drugs (acetaminophen and diclofenac). The signs and symptoms of poisoning disappeared within 36 h post exposure, except for dyspnea in patients 1 and 2. Patients were discharged within 72 h from the vapor’s exposure. Patients 1 and 2 still showed dyspnea and fatigue even after three months. Chemical analysis (liquid chromatography associated with mass spectrometry, LC/MS) on a specimen collected from the aquarium of the poisoned patients revealed high levels of PLTX and 42-hydroxy-PLTX (1018 µg and 46 µg/g wet coral, respectively) [56].

A poisoning probably due to inhalational exposure to PLTXs during soft corals handling has been recently described in Alaska [57]. The case involved three persons: (*i*) patient A who transferred 32 kg of live corals into a 758 L aquarium; (*ii*) patient B and (*iii*) patient C who were asleep in a room adjacent to the aquarium during the coral transfer. During this operation, several coral fragments felt on the floor causing the breaking off of some soft corals. After 7 h, all the patients experienced respiratory (dyspnea, scratchy throat), muscular (myalgia and spasms), neurological (paresthesia, ataxia, weakness, tremors), and gastrointestinal (nausea) symptoms. Patient A, who slept for 7 h in the room with the aquarium, showed the most serious symptoms, including cough, nausea, headache, muscle, and joint pain. At the hospital admission, he was tachycardic, tachypneic, and febrile (maximum temperature: 39.4 °C), with leukocytosis (13.8 × 10^3^ cells/µL and 86% neutrophils. Since the hematochemical parameters, the renal function and chest radiography were within the normal range, the pharmacological treatment was only supportive. The patient A’s symptoms disappeared within two days, whereas patients B and C, reporting less severe symptoms, relapsed in 12 h. Genetic analysis on a soft coral sample, collected from the same home aquarium, identified the species as *Palythoa heliodiscus*. HPLC analysis of the same specimen quantified 7.3 mg PLTX equivalents/g wet weight of zoanthid and LC/MS confirmed PLTX as the main toxin [57].

#### 2.2.2. Cutaneous Exposure to Palytoxins-Contaminated Soft Corals

Two human cases of adverse effects associated to skin exposure to PLTX-contaminated soft corals are documented (Table 2). One of the first reports on adverse effects by cutaneous exposure to soft corals contaminated by PLTXs was anecdotally reported in the early 1960s during the second collection of *Palythoa toxica* in Hawaii, from which PLTX was firstly purified [1]. Collecting the zoanthid colonies with bare hands and feet, a researcher experienced dizziness, nausea, headache, increasing malaise and discomfort to the hands. These symptoms, probably facilitated by small cuts and abrasions caused by the coral collection, lasted for one week and needed supportive pharmacological treatments and medical attention to the feet. Nuclear magnetic resonance (NMR) analysis of *P. toxica* specimens collected during this episode determined a concentration of 280 µg PLTX/g zoanthid [1].

Another case, described in 2008 by Hoffmann and coworkers [58], involved a man in Germany who collapsed 16 h after handling several zoanthid colonies (*Palythoa* sp. and *Parazoanthus* sp.) in his home aquarium. Symptoms started 2 h after contact with the cnidaria and included shivering, myalgia, and general weakness of the extremities. Dizziness and speech disturbance were experienced at the time of collapse. At the admission to the hospital (20 h post exposure), the man showed minor cuts on three fingers, with local inflammatory signs (swelling and erythema). The numbness and paresthesias of the fingers extended to the whole arm over the following 20 h. Hematochemical analyses demonstrated slightly elevated serum levels of CPK (198 IU/L), lactate dehydrogenase (LDH, 304 IU/L), and CRP (13.8 mg/L), consistent with the developed myalgia and skeletomuscular damage. Despite cardiovascular examination revealed a rhythmic heartbeat (83 beats/min) without murmurs and a blood pressure within the normal range (100/70 mmHg), an abnormal electrocardiogram (sinus rhythm of left type with an incomplete right bundle block) was recorded. After intravenous physiological fluids infusion, cardiac signs receded within the next 24 h, but paresthesia, weakness, and myalgia persisted until discharge, 48 h later. Samples of two zoanthid colonies from the aquarium, identified as *Palythoa* sp. and *Parazoanthus* sp., were subsequently analyzed by the hemolysis neutralization assay, detecting high levels of PLTX only in *Parazoanthus* sp. (7700 hemolytic units/g, corresponding to 2–3 mg PLTX equivalents/g wet weight).

### 2.3. Human Poisonings Ascribed to Palytoxins-Contaminated Soft Corals: No Direct Identification of PLTXs in the Corals

A great number of cases describing human poisonings tentatively associated with zoanthids known to produce PLTX-like compounds is reported in the web. The majority of these cases are anecdotally described in aquarium hobbyist forums and blogs, so that it seems quite well known among the aquarists that some corals could be highly toxic. However, no specimen from the involved corals was analyzed for PLTXs, whose involvement had been hypothesized only on the basis of symptoms and the coral species as causative agent of poisoning. Among all these cases, those documented by the scientific literature (data sources: electronic databases PubMed, Scopus, ToxLine, and the references of identified articles) will be discussed.

#### 2.3.1. Inhalational Exposure to Dust or Vapors from Soft Corals

Different cases of adverse effects after inhalation of steam or dust from soft corals were reported, without confirmation of PLTX presence in these cnidarian (Table 1). The first case tentatively ascribed to PLTX inhalation from soft corals was anecdotally reported by Moore *et al.* [1]. In 1961, investigating a soft coral identified as *Palythoa carribaeorum* at the University of Oklahoma, three students experienced chills, nausea, and headache after pulverizing the sun-dried coral in a mixer. The symptoms, probably due to the coral dust leaked out into the atmosphere, resolved within one day. Although no experimental details were given, subsequent coral analysis identified a compound identical to PLTX found in *P. toxica* and *P. tuberculosa* [1].

In 2008, a 32-years-old man, without any previous medical history including asthma or other respiratory diseases, recurred to the Emergency Department in New York (USA) after attempting to eradicate an infesting *Palythoa* coral from his aquarium with boiling water. The coral secreted a mucous-like substance and, immediately after inhaling the foul odor steam, the man experienced shortness of breath and chest pain. At the admission to the hospital, despite normal vital signs, he had wheezing in all lung fields and ECG showed sinus tachycardia (110 beats/min), with no ST-T wave changes and normal QRS and QTc intervals. His respiratory symptoms improved with three doses of the nebulized bronchodilator albuterol, but the chest pain persisted. Since the subsequent clinical analyses did not show elevated cardiac enzymes, dysrhythmia, or other sequelae, the patient was discharged 24 h later. No chemical analyses were performed to identify PLTXs in the coral [61].

Four years later, a poisoning involving four members of a family (a 37-years-old man, his 35-years-old wife, and their two 10-years-old twins) was described in The Netherlands. After attempting to eradicate a zoanthid colony by boiling water, all the patients developed almost the same symptoms (fever, hypotension, nausea, headache, shivering, and severe muscle cramps). After the admission to the emergency room, all of them showed low blood pressure, fever >38.5 °C, leukocytosis, and elevated blood levels of CRP. All the family members recovered within 48 h, after supportive therapy. Although no analyses were carried out to identify the zoanthid species or the presence of PLTX-like compounds, the authors hypothesized a PLTX poisoning [62].

In the same period, a poisoning involving three persons (two men and one woman aged between 21 and 23 years) was described in Switzerland. Within a few minutes after soft coral introduction in their aquarium, they developed dyspnea at rest, dry cough, nausea, headache, fever, and chills. The patients were admitted to the hospital 2 h later and, considering their overlapping symptoms, the clinical picture of the 23-years-old man was described as representative for the case report. His blood pressure was normal and heart and respiratory rates were 121 beats/min and 25 breaths/min, respectively. Arterial oxygen saturation breathing room air was 93% and body temperature was 40 °C. The patient showed a severe hypoxemia (pH 7.36, PaO_2_ 5.6 kPa, PaCO_2_ 6.4 kPa), leukocytosis and mild increase of LDH, CRP, and procalcitonin serum levels. Two days post exposure, fever persisted, and blood inflammatory parameters further increased (CRP = 193.3 mg/L; procalcitonin = 12.82 ng/m; leukocytes = 27.6 × 10^3^ cells/µL). Since the respiratory symptoms worsened on day two, high-resolution computed tomography of the chest was performed, showing zones of patchy and pleural-based consolidation at both lung bases. A pulmonary function test carried out three days after exposure showed a restrictive ventilatory pattern with a normal diffusion capacity, while flexible bronchoscopy revealed a diffuse bronchial swelling with clear bronchial secretion. The broncho-alveolar lavage was slightly turbid, with an elevated cells count (705 × 10^3^ cells/µL), and a predominant granulocytic infiltration pattern (alveolar macrophages = 46%; neutrophils = 49%; lymphocytes = 2%; eosinophils = 3%). All the patients were discharged four days after the poisoning but lung function tests returned within the normal range only at the follow-up visit two weeks later. The soft coral tentatively responsible of the poisoning belonged to the genus *Palythoa*, but no chemical analyses were performed to confirm the presence of PLTX-like compounds [63].

Another case of poisoning ascribed to inhalational exposure to vapors from soft corals was reported by Sud and co-workers [64] and subsequently deepened by Rumore and Houst [65]. It involved five persons: a professional fish tank cleaner, the owner of a fish tank, his wife, and their two children. Immediately after cleaning the fish tank (probably containing *Palythoa* corals) by boiling water, the 42-years-old fish tank cleaner experienced shortness of breath and a body temperature of 38 °C. Six hours later, he was admitted to the emergency room, with increased temperature and leukocytosis (16 × 10^3^ cells/µL). The patient was discharged, with bronchodilator (albuterol) and antibiotic (levofloxacin) therapy. The 51-years-old fish tank owner and his 35-years-old wife, both in proximity of the tank during its cleaning, presented the same symptoms: dry cough developed shortly after the exposure, chills, myalgia, fatigue, fever, vomiting, and paresthesia at the upper extremities. They recurred to the emergency room 8 h after the exposure where, after showering, dry cough, myalgia, and fatigue persisted during hospitalization (three days). Hematology and blood chemistry analyses showed elevated white blood cells (>16 × 10^3^ cells/µL) and a mild increase of LDH (292 and 193 IU/L) and CPK (184 and 197 IU/L),which normalized at hospital day three. The woman, who was two months postpartum, was advised to avoid breast-feeding during her hospital stay. Additionally, the two children were intoxicated. In particular, the three-year-old boy, who was playing close to the fish tank during the cleaning, developed dry cough, an episode of non-bilious and non-bloody emesis, and became fatigued. He was admitted to the emergency room with his parents 8 h after exposure, with fever, high respiratory frequency, and tachycardia (154 beats/min). The child also showed a marked leukocytosis (35.4 × 10^3^ cells/µL) and elevated blood levels of LDH (331 IU/L). The two-month-old girl, the farthest from the fish tank, was asymptomatic but presented a marked leukocytosis (34.4 × 10^3^ cells/µL) and high levels of LDH (507 IU/L), venous lactate (5 mmol/L), CPK (259 IU/L) and creatine kinase MB isoenzyme (CKMB; 7.82 ng/mL). An episode of constipation occurred when she was switched back to the breast milk. Both children received hydration therapy and were discharged within 48 h. In this case, no analyses were performed to detect PLTXs in the patients serum or tissues or in the corals.

A series of adverse effects associated to inhalation of vapors from soft corals was recently reported in Alaska by an aquarium shop owner, in collaboration with the Alaska Section of Epidemiology (SOE). The first poisoning involved two persons admitted to the intensive care unit after cleaning a fish tank containing zoanthids by hot water to remove polyps from a rock base. Both the persons experienced fever, tremors, weakness, and ataxia. The first patient, a pregnant female, was subjected to a preterm labor the day after her hospital admission and gave birth her baby at six months’ gestational age. The second patient presented persistent pulmonary symptoms even after two years. Additionally, their dog showed symptoms of poisoning (vomit and lethargy). The second poisoning occurred in July 2014, when seven aquarium shop staff members dismantled a private aquarium and handled corals in the shop. Four of them, interviewed by the SOE, experienced a bitter metallic taste after the inhalation of hot water vapors, followed by cough, joint and muscle pain, fever, tremors, and weakness. All these symptoms mainly resolved within the following morning. Several weeks after, two staff members referred similar symptoms after handling the same corals and cleaning some aquarium components with hot water. In these cases, no analyses for zoanthid identification or PLTXs detection were carried out [57].

Another case involving three members of a family was recently described in New York. A 53-years-old man, with pre-existing hypothyroidism, hyperlipidemia, psoriasis, and smoking history, presented to the emergency room with his wife and their daughter, about 8 h after cleaning an exotic coral from his home aquarium with hot tap water without using any protective equipment. The coral was described by the patient as a species of Zoantharia. The man experienced the first symptoms 1–2 h after exposure to the vapors, including fever, chills, myalgia, and dyspnea. His wife and his daughter, which were in adjoining basement room or upstairs in the first floor, presented the same but less severe symptoms and they were discharged 24 h after admission. The man’s clinical examinations revealed tachycardia (112 beats/min), a blood pressure of 155/83 mmHg, and a respiratory rate of 18 breaths/min and fever (39 °C). Laboratory analyses showed a leukocytosis (14 × 10^3^ cells/µL). He was treated with acetaminophen and nebulized albuterol, with minimal improvement of symptoms. The man conditions worsened needing supplemental oxygen, repeated doses of nebulized albuterol and oral acetaminophen. The patient was transferred to intensive care unit where he experienced worsening cough, increased generalized weakness, and malaise. On the second in-patient day, he developed hemoptysis, while serial chest X-rays showed worsening bibasilar opacities. After three days of hospitalization, leukocytosis increased up to 22 × 10^3^ cells/µL. By the day four, the patient’s conditions improved but supplemental oxygen by face mask was still necessary. After seven days the man was discharged with portable oxygen, an albuterol metered-dose inhaler, and prednisone taper. He completely recovered one month after discharge. No analyses to identify the coral or to detect PLTXs were carried out [66].

#### 2.3.2. Cutaneous Exposure to Soft Corals

Only two cases of adverse effects after cutaneous exposure to zoanthids, without confirmatory analyses of PLTX involvement, are documented (Table 2). In the first case, a 25-years-old healthy woman, with intact skin, developed both cutaneous and systemic signs and symptoms of poisoning after handling a zoanthid in a home aquarium without any barrier protection, such as gloves and goggles. She experienced a metallic taste, followed by perioral paresthesia and hives on her torso and extremities. Paresthesia and dysguesia resolved the day after exposure, but the upper lip was edematous. The woman, admitted to the emergency room two days after exposure, presented increased edema and pruriginous erythema at both hands, with normal sensory and motor functions, but also urticarial rashes at bilateral upper arms, thighs, abdomen, upper chest, and back. As rashes appeared to be histamine-mediated, the patient was treated with intravenous diphenhydramine (50 mg) in addition to supportive corticosteroid (methylprednisolone, 125 mg) and benzodiazepine (lorazepam, 1 mg). Once the woman’s symptoms were resolved, she was discharged with five days’ oral therapy with prednisone (40 mg) and diphenhydramine (50 mg). Also in this case, zoanthid identification and PLTXs detection were not performed [67].

The second case occurred in Georgia, where a marine aquarium hobbyist recurred to the hospital after skin contact with a zoanthid coral. Immediately after exposure, he experienced chest pain, lightheadedness, weakness and numbness on the left arm. At the admission, the patient presented elevated heart rate and blood pressure, high blood levels of CPK and sinus tachycardia. The chest pain and the numbness of the arm lasted up to 4 h after admission, while elevated CPK persisted even 16 h later. In this case, hemolytic neutralization assay was carried out on a patient serum sample collected 1 h after exposure. The serum exerted an hemolytic activity but, since it was not neutralized by an anti-PLTX antibody, the hemolytic agent was not confirmed as PLTX. Accordingly, HPLC and LC/MS analyses on the serum sample did not identify any detectable compound consistent with standard PLTX. These evidences suggest that, if a PLTX-like compound was the causative agent of poisoning, the analyzed serum might contain possible PLTX metabolite(s) and/or PLTX analogue(s) not detectable by the anti-PLTX antibody [54].

#### 2.3.3. Ocular Exposure to Soft Corals

Three cases of adverse effects by ocular exposure to soft corals were documented and summarized in Table 3. Two cases were described in United States by Moshirfar *et al.* [59]. In the first case, a 31-years-old man, who wore soft contact lenses, developed ocular irritation and redness, as well as a bitter metallic taste immediately after drilling a zoanthid coral from a rock in his saltwater aquarium. The day after exposure, the man had a severe eye pain, eyelid swelling, photophobia, and a purulent discharge from both eyes. Specific eye examination revealed maximum visual acuity, with diffuse bilateral punctate epithelial erosions. Thus, the patient was topically treated with 0.5% moxifloxacin and artificial tears. Three days after exposure, his condition worsened: the ocular pain increased, the visual acuity declined and he presented a significant conjunctival hyperemia, punctate epithelial erosions in the right cornea and a central epithelial defect with a stromal ring infiltrate in the left eye. Hence, 1% prednisolone acetate (three times a day), 0.1% fluorometholone ointment (at bedtime), 0.5% moxifloxacin drops (four times a day), and oral doxycycline and ascorbic acid were prescribed. However, no significant improvement of the left eye’s condition was observed. Thus, 1% prednisolone acetate drops were increased to hourly doses and a therapeutic contact lens was applied. On resolution of the epithelial defect, fluoromethoxolone and moxifloxacin treatments were discontinued, and the prednisolone acetate drops were tapered over sixe weeks. The treatment with 0.05% cyclosporine drops, twice a day for 12 weeks, also resolved the stromal ring infiltrate but a 30% stromal thinning remained in the midperiphery, associated with central steeping. The second case involved a 49-years-old man, who had undergone laser eye surgery in both eyes, four years previously. Handling zoanthid corals without gloves, he accidentally rubbed his right eye, experiencing ocular pain, redness, and blurry vision. After one day, the man presented a defect on visual acuity, a papillary reaction of the upper and lower palpebral conjunctiva, a bulbar conjunctival injection, and punctate epithelial erosions of the cornea. After a topical treatment with 0.5% moxifloxacin and 1% prednisolone acetate drops, all symptoms resolved with maximum visual acuity recovery. In both cases, no analyses to identify the coral and to detect palytoxin were carried out [59].

Recently, a case of ocular exposure to a splash from a zoanthid referred as “encrusting anemone” involved a 63-years-old man in Switzerland. Handling the zoanthid out of water, an accidental splash into the right eye induced a local burning sensation. The man rinsed the eye with tap water for several minutes before trying a treatment with chamomile and black tea. Two and a half hours after exposure, the patient recurred to the Emergency Unit due to dyspnea, nausea and shivering. Upon admission, laboratory analyses showed a high white blood cell count (20.29 × 10^3^ cells/µL) and CRP (47.4 mg/L), in addition to slightly elevated serum levels of CPK (261 IU/L) and LDH (267 IU/L), and positive urine for myoglobin (39 µg/L). These findings led to suppose a rhabdomyolysis, which required monitoring in the intensive care unit and intravenous infusion of balanced crystalloid solution. On the second day, the laboratory parameters improved and the patient was discharged. Ocular examination showed a marked conjunctival injection and superficial punctate epitheliopathy in both the eyes. The right eye was more affected, with a multiple corneal Descemet’s folds and pH elevated to 8.5, subsequently reduced to 7.5 by rinsing with a washing solution (Diphoterine^®^). The man’s visual acuity in both eyes was reduced to counting fingers. A therapy with antibiotic (0.5 moxifloxacin) and steroid (1% prednisolone acetate) drops was immediately started. On the second day, the eyes lesion worsened: both eyes showed incomplete corneal erosions and anterior chamber reactions, the right eye being most affected, as well as Descemet’s folds and partially avascular conjunctiva. Despite the topical antibiotic and steroid treatment, the corneal erosions did not heal and ulcers developed one week after exposure. Then, amniotic membrane transplantations were performed on both eyes. The cornea healed after several weeks, with residual thinning, while the remaining irregular corneal astigmatism was corrected four months later fitting scleral contact lenses. Then, visual acuity recovered to 0.8 in the right eye and 1.0 in the left eye [60].

As mentioned above, the major human poisonings ascribed to PLTX-contaminated soft corals are described anecdotally in aquarium hobbyist web forums. Several cases are ascribed to ocular exposure to soft corals, but a correct identification of the involved zoanthids is often missing. Only in a few cases, the scientific (species or genus) or the common coral’s name, such as *Zoanthus gigantus*, *Palythoa singaporensis*, *Protopalythoa* sp., and *Zoanthus* sp., are known. Considering the different online marine aquarium forums, 15 cases of ocular exposure to soft corals tentatively containing PLTXs can be found. In most cases, signs and symptoms of poisoning, often consequent to handling soft corals outside the water, were pain, redness, and swelling. Seven patients experienced corneal inflammation and ulceration with partial loss of vision, while only in three cases systemic symptoms, such as nausea, fever, and shivering, were reported. The clinical course, the duration of symptoms, and the treatment were always unknown, except for few cases in which symptoms resolved between four days and seven months after exposure. Signs and symptoms were often treated rinsing the eyes with water, administering artificial tears, steroids drops, and/or antibiotics, while in one case the patient used contact lenses for four years after exposure to correct the vision [60].

### 2.4. Pharmacological Treatments

No antidote has been developed against PLTX-like compound poisonings, so far. Consequently, no defined and harmonized medical protocols for the treatment of PLTX poisonings have been established. In general, pharmacological treatments are symptomatic, being optimized to reduce or limit the signs and symptoms of poisoning. Therefore, they are defined case by case.

Nevertheless, pharmacological treatments in human poisonings ascribed to PLTX-contaminated soft corals are quite detailed. Regarding poisonings due to inhalational exposure, different approaches have been described but consisted mainly in nebulized β-agonists or corticosteroids and/or systemic corticosteroids. Alternatively, other pharmacological approaches were based on associations of non steroidal anti-inflammatory drugs (NSAIDs) and nebulized β-agonists, or corticosteroids and histamine antagonists combinations [2]. Adverse effects by cutaneous exposure are usually treated with supportive intra-venous physiological fluids infusion and associations of corticosteroids and antihistamines. Similarly, the signs and symptoms of poisoning by ocular exposure are treated with artificial tears and corticosteroids. In the latter cases, surgical interventions, such as amniotic membrane transplantations, could be the resolving therapeutic option in case of severe eyes damages, such as keratolysis and ulcers.

In general, it has to be noted that these therapies are usually associated with antibiotics. This approach could be useful only when the etiology is uncertain but, it is worthless when the poisonings are certainly due to PLTXs.

## 3. Palytoxins in Soft Corals

### 3.1. Palytoxin Analogs Identified in Soft Corals

PLTX was firstly isolated from the Hawaiian soft coral *Palythoa toxica* [1] but it was detected also in other species belonging to the genus *Palythoa* (Table 4), such as *P.* aff. *margaritae* [68], *P. vestitus* from Hawaii [69], *P. mammillosa* and *P. caribaeorum*, both collected from the coral reefs of the Caribbean Sea [3,70,71,72]. Intriguingly, PLTXs were also identified in soft corals cultured as decorative elements in home aquaria [54,58]; characteristic is the case of *P. heliodiscus* [55,56,57]. In addition, PLTX has been identified in other zoanthids belonging to the genera *Zoanthus* and *Parazoanthus*, growing in colonies close to those of *Palythoa* in the coral reef. Additionally, in this case, PLTX was identified both in the wild corals (*Z. sociatus* and *Z. soladeri*) [3] and in corals cultured in home aquaria [58].

Furthermore, a series of PLTX analogs have been identified in *Palythoa* soft corals (Table 4). The firstly identified analogs were homo-PLTX, bis-homo-PLTX, neo-PLTX, and deoxy-PLTX, detected together with PLTX in *P. tuberculosa* [73,74,75]. Subsequently, a 42-hydroxy derivative of PLTX (42-OH-PLTX), which structure was characterized as a 42*S*-OH-50*S*-PLTX, was identified in *P. toxica* [14]. In addition, a stereoisomer of this analogue with a conformational inversion at C50 (42*S*-OH-50*R*-PLTX) was identified in *P. tuberculosa* [15].

It has to be noted that among the different genera of soft corals used as decorative elements in home aquaria (*i.e.*, *Palythoa*, *Zoanthus*, *Sarcophyton, Sinularia, Nephthya, Cladiella*, and *Xenia*) [76,77], the *Palythoa* and *Zoanthus* genera are frequently sold without any precaution or information about their toxicity [55,78], even though many specimens may contain high levels of PLTXs. For instance, four out of five samples of *P. heliodiscus* purchased from an aquarium store in Maryland (USA) contained high amounts of PLTX (from 613 to 1164 µg/g) and one sample contained mainly deoxy-PLTX (3515 µg/g) [55].

**Table 4:** PLTX and its analogs identified in Palythoa and Zoanthus soft corals.

Genus	Species	Toxin	Coral Origin	Detection Method	References
*Palythoa*	*toxica*	PLTX	Coral reef; home aquarium *	NMR	[1]
		42*S*-OH-50*S*-PLTX	Coral reef; home aquarium *	LC/MS, NMR	[14]
	*tuberculosa*	PLTX	Coral reef	Mouse bioassay, HPLC, HPTLC, UV detection	[73,74,75]
		42*S*-OH-50*R*-PLTX	Coral reef	HR LC/MS, NMR	[15]
		Deoxy-PLTX	Coral reef	HPLC	[73]
		Homo-PLTX			
		Bis-homo-PLTX			
		Neo-PLTX			
	*vestitus*	PLTX	Coral reef	N.D.	[69]
	*margaritae*	PLTX	Coral reef	HPLC, NMR	[68]
	*mammillosa*	PLTX	Coral reef	Ion exchange chromatography, haemolysis neutralization assay, HPLC	[3,70]
	*caribaeorum*	PLTX	Coral reef	Ion exchange chromatography, haemolysis neutralization assay, HPLC	[3,9,70,71,72]
	*heliodiscus*	PLTX	Home aquarium	HPLC, ESI-LC/MS	[55]
		Deoxy-PLTX			
		42-OH-PLTX **	Home aquarium	LC/MS	[56]
	*caesia*	PLTX	Coral reef	Haemolysis neutralization assay, HPLC	[72]
	N.D.	PLTX	Home aquarium	Haemolysis neutralization assay	[58]
*Zoanthus*	*sociatus*	PLTX	Coral reef	Haemolysis neutralization assay, HPLC	[3]
	*soladeri*	PLTX			
	*pulchellus*	PLTX		Haemolysis neutralization assay	[9]

* Unpublished results; ** Structural conformation at C50 was not reported; N.D. = not determined.

### 3.2. Toxicity of Palytoxin Analogs Identified in Soft Corals

Toxicological studies on PLTX analogs identified in soft corals are limited to 42-OH-PLTXs. In the first study, the acute oral toxicity of 42*S*-OH-50*S*-PLTX was characterized in mice [79]. After single oral administration of 42*S*-OH-50*S*-PLTX in mice (300 to 1697 µg/kg), the LD_50_ (median lethal dose) was 651 µg/kg (95% confidence limits, CL = 384–1018 µg/kg), comparable to that of PLTX (LD_50_ = 767 µg/kg; 95% CL = 549–1039 µg/kg), as reported by Sosa and coworkers [80]. Following the toxin administration, animals showed progressive signs and symptoms of toxicity, such as scratching, jumping, paralysis of the hind limbs, respiratory distress, and cyanosis, similar to those observed after acute oral administration of PLTX [80]. Mice that spontaneously died within 24 h showed tissue alterations in the pancreas (decreased exocrine secretions in the acinar lumen) and liver (hepatocellular glycogen content). In addition, alterations at the non-glandular stomach (focal inflammatory lesions involving mucosa, submucosa and muscularis externa) were observed after 24 h, at doses ≥424 µg/kg. Hematochemistry showed a marked increase of LDH and aspartate-aminotransferase (AST) at doses ≥600 µg/kg, besides alanine-aminotransferase (ALT), CPK and K^+^ at doses ≥848 µg/kg. Although no significant histological alterations were observed in skeletal and cardiac muscles, the increased concentrations of both AST and ALT, associated with the increased CPK and LDH plasma levels, suggested these tissues as primary targets of 42*S*-OH-50*S*-PLTX, as previously recorded after acute oral administration of PLTX in mice [80].

Hence, to characterize the mechanism of skeletal muscle damage, the effects of 42*S*-OH-50*S*-PLTX were subsequently studied *in vitro* on primary cultures of skeletal muscle cells. Cells exposure to 42*S*-OH-50*S*-PLTX (6 nM) induced an increased intracellular Ca^2+^ level, comparable to that induced by the same PLTX concentration. Similarly, binding affinity of 42*S*-OH-50*S*-PLTX towards a purified Na^+^/K^+^ ATPase (IC_50_ = 29.4 ± 3.1 nM) was similar to that of PLTX (IC_50_ = 28.2 ± 7.0 nM), but the two toxins displayed different sensitivity to ouabain [14]. Moreover, 42*S*-OH-50*S*-PLTX induced an irreversible concentration-dependent cytotoxicity (EC_50_ = 0.30 ± 0.07 nM), comparable to that of PLTX (EC_50_ = 0.54 ± 0.07 nM) [79]. A short exposure to 42*S*-OH-50*S*-PLTX (10 min, 6 nM) induced a marked inhibition of the functional cells response to acetylcholine and an increased cell volume, dependent on extracellular Na^+^. These effects were in agreement with those of PLTX [81,82]. Further Ca^2+^ imaging analysis revealed that 42*S*-OH-50*S*-PLTX concentrations higher than 1 nM, caused a biphasic increase of intracellular Ca^2+^ similar to that induced by PLTX [81]. Moreover, 42*S*-OH-50*S*-PLTX cytotoxicity was abolished by the Ca^2+^-free culture medium only at concentrations lower than 1 nM, indicating a secondary role of Ca^2+^ ions in the toxin cytotoxicity. This result could represent an important biological difference between the cytotoxic pathway evoked by 42*S*-OH-50*S*-PLTX and PLTX at the muscular level [81].

Other *in vitro* studies evaluated the hemolytic activity of this toxin in mice erythrocytes, showing a similar activity between 42*S*-OH-50*S*-PLTX and PLTX (EC_50_ = 7.6 ± 0.5 × 10^−12^ M and 13.2 ± 0.1 × 10^−12^ M, respectively) [79].

Considering the adverse effects associated with the cutaneous exposure to soft corals contaminated by PLTXs, the effects of 42*S*-OH-50*S*-PLTX isolated from *P. toxica* were recently evaluated on HaCaT skin keratinocytes, in comparison to those of its stereoisomer 42*S*-OH-50*R*-PLTX from *P. tuberculosa* [15]. The cytotoxicity of 42*S*-OH-50*S*-PLTX was about one order of magnitude higher than that of its stereoisomer (EC_50_ = 1.0 × 10^−10^ M and 9.3 × 10^−10^ M, respectively), and almost one order of magnitude lower than that of PLTX (EC_50_ = 2.7 × 10^−11^ M). This suggests that even one conformational change in PLTX structure, such as that at C50, can significantly influence the biological activity of these structurally complex molecules [15].

Given the availability of sufficient amounts of purified PLTX analogs, these results should be investigated broader and deeper by *in vivo* studies, to characterize the actual toxicological potential of these toxins, widely found in *Palythoa* soft corals.

## 4. Discussion

Since the 1980s, the popularity of home aquaria containing living corals has been dramatically increased. As a result of the increased trade of live corals over the years, concern has been raised about the impact on human health and the possible adverse effects associated with the manipulation and maintenance of these corals. Indeed, most soft corals found in marine aquaria are collected from the wild and, in home aquaria, they can find the optimal conditions to grow, proliferate and, eventually, accumulate toxic compounds. Among the different species of decorative soft corals, such as *Sarcophyton, Sinularia, Nephthya, Cladiella, Xenia, Palythoa*, and *Zoanthus* species [76,77], those belonging to the last two genera are widely used due to their colorful and ornamental features [55,78]. The latter are known to accumulate PLTX [2,54] and/or its analogs, such as 42*S*-OH-50*S*-PLTX isolated from *P. toxica* [14], 42*S*-OH-50*R*-PLTX identified in *P. tuberculosa* [15], and deoxy-PLTX isolated from *P. heliodiscus* [55].

PLTX-like compounds are largely known for their toxicity. A large number of studies, both *in vivo* [16,79,80,81] and *in vitro* [28,29,30,31,32,33,34,35,36,37,38,39,40,41,42]*,* have been performed in the last three decades to characterize PLTX toxicity and the relevant mechanism of action [25,26,27,33]. PLTX is recognized as one of the most toxic non-proteinaceous natural compound known, so far. Although the most harmful route of human exposure to PLTXs is the oral intake, PLTX toxicity after inhalational and cutaneous exposure represents a serious problem for human health, as well [2]. These exposure routes are the most frequent ones and are involved in human poisonings postulated to PLTX-contaminated soft corals. It is noteworthy that the main signs and symptoms referred after inhalation of hot vapors from aquaria containing soft corals are similar to those reported after inhalational exposure to marine aerosol during *Ostreopsis* blooms. Thus, the similarity of symptoms after inhalational exposure to vapors from aquaria or seawater aerosol suggests the involvement of a common toxic agent, such as PLTXs, as supported by a recent study in which PLTXs were found in marine aerosols during *Ostreopsis* blooms [83]. Similarly, the direct *in vitro* effects of PLTX and PLTX-like compounds, such as 42*S*-OH-50*S*-PLTX and 42*S*-OH-50*R*-PLTX, on skin keratinocytes demonstrate the cutaneous toxicity of these toxins [28,32,34,35], at the basis of the adverse effects ascribed to PLTXs after skin contact to soft corals or to seawater containing *Ostreopsis* cells [2].

Despite the proven toxicity of PLTXs, the risks posed by keeping soft corals in home aquaria are largely unrecognized and underestimated by aquarium hobbyists and stores owners. In addition, these soft corals are widely sold in the aquarium trade without any warning about their toxic potential or guidelines for their use and maintenance. Indeed, the trade of these cnidarians, which may cause severe poisonings with significant sanitary and economic impacts, is still not regulated. Although documented cases of PLTX poisonings due to contact with soft corals in home marine aquaria are limited, they probably represent only the tip of the iceberg. In fact, the entity of the symptoms experienced by poisoned subjects after inhalational, cutaneous, or ocular exposure to soft corals, their secretions or vapors due to hot water poured on these cnidarians do not always require a sanitary intervention. In addition, these poisonings could be attributed to a different etiology. Considering this point, it is extremely important that physicians are properly informed on the adverse effects associated with soft corals exposure in order to diagnose and manage these kinds of poisonings. To this aim, guidelines concerning an harmonized pharmacological protocol to be followed in these cases are required. In this view, we propose a draft chart (Supplementary Material Figure S1) useful for the clinician to document and handle these cases of poisoning. From literature data, the “case definition of PLTXs poisonings ascribed to soft corals” is suggested, together with the main symptoms to be checked and the hematoclinical analyses to be carried out. Furthermore, specific analyses on biological specimens from poisoned patients and on the suspected soft corals are suggested as contribution to define this kind of poisoning.

Similarly, although many marine aquarium hobbyists handled zoantharians for years without any documented PLTX-related poisonings, we strongly believe that complete information for the consumer are necessary. Considering the extremely high toxicity of some *Palythoa* species, the dermotoxicity of PLTXs and PLTX skin tumor promotion [84,85], we advise against handling *Palythoa* with bare hands, recommending the use of protective gloves, glasses, and a breathing mask [58,63]. Since disposable latex or nitrile gloves break easily in contact with sharp stone corals or rocks in aquaria, long robust rubber gloves with protection of the forearms are the most suitable ones. Additionally, the use of boiling water or brushing to kill the soft corals in aquaria can be dangerous and is not recommended. The safest method of their eradication is removing undesirable *Palythoa* colonies along with their substrates from aquaria and throwing them away in a safe condition, while wearing personal protective equipment.

In conclusion, the presence of the highly-toxic PLTX and/or its analogs in soft corals, widely used as decorative elements in marine home aquaria, represents an emerging and still underestimated sanitary problem. Once known only among the aquarium hobbyists, cases of poisoning by inhalational, cutaneous, and/or ocular exposures, sometimes characterized by severe symptoms, are continuously increasing in number, also in the scientific literature. A deeper characterization of these poisonings should be provided in order to gain a complete knowledge from a toxicological and medical point of view.

## Supplementary Files

Supplementary File 1

## Abbreviations

The following abbreviations are used in this manuscript:

ALT: alanine-aminotransferase
AST: aspartate-aminotransferase
CKMB: creatine kinase MB isoenzyme
CL: confidence limits
CPK: creatinine phosphor-kinase
CRP: C-reactive protein
ECG: electrocardiogram
ESI-MS: electrospray ionization-mass spectrometry
HPLC: high performance liquid chromatography
LC/MS: liquid chromatography associated with mass spectrometry
LD50: dose giving 50% of lethality
LDH: lactate dehydrogenase
NCE: Na^+^/Ca^2+^ exchanger
NHE: Na^+^/H^+^ exchanger
NMR: nuclear magnetic resonance
Ost-D: ostreocin-D
OUA: ouabain
OVTX: ovatoxin
PLTX: palytoxin
