# Supplementary material for: Palytoxin-Containing Aquarium Soft Corals as an Emerging Sanitary Problem
Source: Mar Drugs. 2016 Feb 4;14(2):33. doi: 10.3390/md14020033 (PMC4771986; doi:10.3390/md14020033)
Supplement: Supplementary File 1 [file marinedrugs-14-00033-s001.pdf]

Supplementary Materials: Palytoxin-Containing Aquarium Soft Corals as an Emerging Sanitary Problem

Marco Pelin, Valentina Brovedani, Silvio Sosa and Aurelia Tubaro *

Figure A1: Draft chart for palytoxin/s poisonings associated with soft corals

Date………………………….

Identification of the patient (first two letters of Name and Surname) | | |

Date of birth:	Sex:	Date of onset of symptoms:

1. Case Definition:

**Inhalational exposure**	• soft coral eradication by boiling water and/or by brushing of the coral<br>• cleaning of the aquarium using hot water<br>• seeking medical care<br>• presence of at least two of the following signs/symptoms: cough, dyspnea, fever ≥ 38 °C, tachycardia, leukocytosis, and chest pain
**Cutaneous exposure**	• contact with aquarium water in presence of *Palythoa* and/or *Zoanthus* soft corals<br>• skin contact with *Palythoa* and/or *Zoanthus* soft corals<br>• presence of at least two of the following signs/symptoms: erythema, edema, paresthesia, ECG alterations
**Ocular exposure**	• eye contact with *Palythoa* and/or *Zoanthus* soft corals' secretions<br>• eye contact with hands after *Palythoa* and/or *Zoanthus* soft corals manipulation<br>• presence of at least two of the following signs/symptoms: ocular irritation, punctuate epitheliopathy, photophobia, conjuctival hyperemia

	**Inhalational Exposure**	**Cutaneous Exposure**	**Ocular Exposure**	**Other**
Location of exposure				
Date of exposure				
Duration of exposure				
Latency btw onset of symptoms and exp				

2. Signs and Symptoms

	YES	NO		YES	NO
Fever ≥ 38 °C			ECG alterations		
Cough			Erythema		
Headache			Edema		
Dyspnea			Paresthesia		
Sore throat			Myalgia		
Rhynorrhea			Ocular irritation		
Chest pain			Photophobia		
Tachycardia			Conjunctivitis		

Pre-Existent Pathologies and Medical Conditions:

Asthma and other chronic pulmonary disease   Dermatitis
Rhinitis   Ocular pathologies (specify)
Seasonal allergies   Contact lenses
Influenza   Other (specify)
Cardiovascular diseases (specify)

Drugs used at the moment of the exposure or before

3. Diagnostic Examinations

Blood	YES (Indicate Values and Lab-Range)	NO
Blood Count		
Leukocyte count		
CPK		
LDH		
AST		
ALT		
C-reactive protein		
Na^+		
K^+		
…………..		
…………..		

URINE	YES (Indicate Values and Lab-Range)	NO
Color		
Myoglobinuria		
K^+		
………………		
………………		

Other	YES (Indicate Observations)	NO
ECG		
Chest X-rays		
……………………….		
……………………….		

4. Therapy

……
……
……

5. ACCESS to Emergency Department NO YES

Hospitalization NO YES
Day of admission: …………
Day of discharge: …………

Time after exposure: ………............................ Time of recovery: ....

6. Laboratory Analysis for Pltx/s Identification

Raw material for analysis: ……………………………………………………………………………….
If possible, please specify the soft coral species.……………………………………………………………………

	Soft coral	Aquarium water	Other *
HPLC analysis			
LC/MS analysis			
Hemolysis neutralization assay			
ELISA method			
……..			

* Please specify…………………………………......…………………………………………..

7. Notes

………
………
………
